# Safety and immunogenicity of an adjuvanted human onchocerciasis vaccine candidate, *Ov* MANE1: preclinical evaluation in a mice model

**DOI:** 10.1002/cti2.70114

**Published:** 2026-06-30

**Authors:** Derrick Neba Nebangwa, Mary Teke Efeti, Sonia M. E. Momnougui, Cabirou Mounchili Shintouo, Robert Adamu Shey, Rene Bilingwe Ayiseh, Fidele Ntie‐Kang, Stephen Mbigha Ghogomu

**Affiliations:** ^1^ Department of Biochemistry and Molecular Biology, Faculty of Science University of Buea Buea Cameroon; ^2^ Center for Drug Discovery University of Buea Buea Cameroon; ^3^ CoVACC, Community‐Led Vaccination Yaoundé Cameroon; ^4^ Frailty in Ageing Research Group Vrije University Brussels Brussels Belgium; ^5^ Department of Gerontology, Faculty of Medicine and Pharmacy Vrije University Brussels Brussels Belgium; ^6^ Graduate School of Life Sciences Julius‐Maximilians University WÜrzburg WÜrzburg Germany; ^7^ Department of Microbiology and Immunology, College of Medicine Drexel University Philadelphia Pennsylvania USA; ^8^ Drugs and Molecular Diagnostic Laboratory, Biotechnology Unit University of Buea Buea Cameroon; ^9^ Department of Chemistry, Faculty of Science University of Buea Buea Cameroon; ^10^ Institute of Pharmacy Martin‐Luther University of Halle‐Wittenberg Halle Germany

**Keywords:** antibody and cellular immune response, BALB/c mice, chimeric antigen, onchocerciasis, *Ov*MANE1, vaccine

## Abstract

**Objectives:**

Onchocerciasis, caused by the filarial worm *Onchocerca volvulus*, remains a major public health challenge because of the limitations of ivermectin‐based control strategies, thereby highlighting the need for more innovative tools such as vaccines.

**Methods:**

This study investigated the safety and immunogenicity of a novel multi‐epitope chimeric antigen, *Ov*MANE1 formulated with Freund's adjuvant, in BALB/c mice. Mice were immunised at three time points of 2‐week intervals, with blood collected at each point to measure antibody levels and immune cells.

**Results:**

Adjuvanted‐*Ov*MANE1 exhibited a promising safety profile, revealing neither any physical signs of toxicity nor behavioural abnormalities. Immunological assays showed significant increases in total IgG absorbance (OD 450 nm) after the first (*P* = 0.0260) and final booster doses (*P* = 0.026). Total IgG (*P* = 0.0086) and IgG1 (*P* = 0.0465) absorbances also increased significantly over the study period, suggesting sustained humoral immunity. Moreover, cellular responses were significantly enhanced, with elevated leucocyte count (*P* = 0.0190) and increased lymphocyte activity (*P* = 0.0397) observed in the adjuvanted‐*Ov*MANE1 group compared to the control. Indeed, total leucocytes increased progressively from Day 0 to Day 39, with significant differences recorded in the test group between doses: Days 0–14 (*P* = 0.0043) and Days 14–28 (*P* = 0.0079). The pronounced production of relevant antibodies and induction of cellular immunity is indicative of a mixed Th1/Th2 response and antibody‐dependent cellular cytotoxicity (ADCC) targeting *O. volvulus* L3 and/or other larval stages of the parasite.

**Conclusion:**

These results position *Ov*MANE1 as a promising vaccine candidate against human onchocerciasis. However, further *in vivo* studies are needed to confirm and expand these findings.

## Graphical abstract

*Ov*MANE1 formulated with Freund's adjuvant was safe and elicited strong humoral and cellular immune responses, including significant IgG and leukocyte activation. These findings highlight *Ov*MANE1 as a promising multi‐epitope vaccine candidate for advancing onchocerciasis prevention.

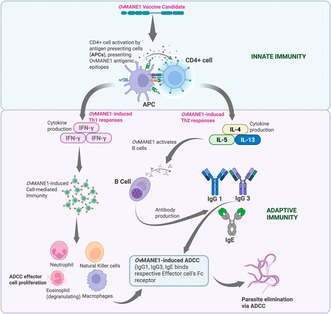

## Introduction

Human onchocerciasis, commonly known as river blindness, is one of the most devastating yet neglected tropical diseases (NTDs), caused by the filarial nematode *Onchocerca volvulus* through exposure to repeated bites of an infected *Simulium* blackfly that breeds around fast flowing rivers.^1^, ^2^ Onchocerciasis primarily manifests clinically through severe itching, disfiguring skin conditions and visual impairments including permanent blindness, which has led to its recognition as the second leading infectious cause of global blindness, after trachoma.^2^ Currently, *O. volvulus* is endemic in Africa, Central America and South America with > 99% of all cases occurring in sub‐Saharan Africa.^1^ According to the 2017 Global Burden of Disease Study, 14.6 million infected individuals were reported to have skin disease, while 1.15 million suffered from vision loss.^3^ Recent WHO 2024 statistics report at least 249.5 million people in 28 countries requiring interventions to eliminate onchocerciasis with 18.8 million people needing preventive chemotherapy (PC).^2^ Because of its substantial impact on health, onchocerciasis has been a long‐standing priority for international disease control groups such as the Onchocerciasis Elimination Program for the Americas (OEPA) and the African Programme for Onchocerciasis Control (APOC). However, control efforts have primarily relied on two strategies: combating the *Simulium* fly vector through aerosol spraying and administering ivermectin as large‐scale chemotherapy. While both methods have been used individually or in combination, these control strategies face several limitations.^4^ These include insecticide and ivermectin resistance, high costs and logistical challenges, lack of ivermectin macrofilaricidal activity, ineffectiveness of sprays in certain areas and the re‐infestation of forests by black flies, hindering the complete elimination of onchocerciasis.^5^, ^6^ Moreover, the restriction on ivermectin usage in areas where onchocerciasis and loiasis co‐exist poses one of the most significant bottlenecks to onchocerciasis control.

Earlier in 2015, an international consortium launched a new global initiative, named The Onchocerciasis Vaccine for Africa (TOVA) with the key goal to advance the development of vaccine candidates meeting the desired target product profiles (TPP) for an onchocerciasis vaccine.^7^ The TPP targets a preventive vaccine for children under the age of 5 who are restricted from taking ivermectin, or a therapeutic vaccine for both adults and children infected with onchocerciasis. Lustigman *et al*. (2018) reported TOVA's process that led to the selection of several protein sub‐unit vaccine candidates for further clinical development based on proven efficacy both *in vitro* and in animal models.^8^, ^9^, ^10^, ^11^, ^12^ However, these antigens offer only partial protection.^9^, ^13^ Moreover, issues such as immune evasion, especially when addressing pathogens with immunomodulatory properties such as *O. volvulus*,^14^ frequently lead to unsatisfactory results in human proof‐of‐concept clinical trials. Thus, there remains a dire need for more research addressing the design and development of more efficacious vaccines. Multi‐epitope vaccines containing both B‐ and T‐cell epitopes, covering various antigens expressed across multiple larval stages of parasitic worms have been suggested as innovative strategies for combating filarial infections.^15^ These vaccines offer significant advantages, including the ability to induce a balanced Th1‐ and Th2‐specific protective immune response, as well as risk minimisation of adverse effects associated with unfavorable epitopes present in a full antigen sequence or whole parasite‐based vaccines.^16^, ^17^ Novel multi‐epitope vaccine candidates have demonstrated promising efficacy against other nematode infections, including lymphatic filariasis and trichinellosis.^18^, ^19^

**Figure 1:**
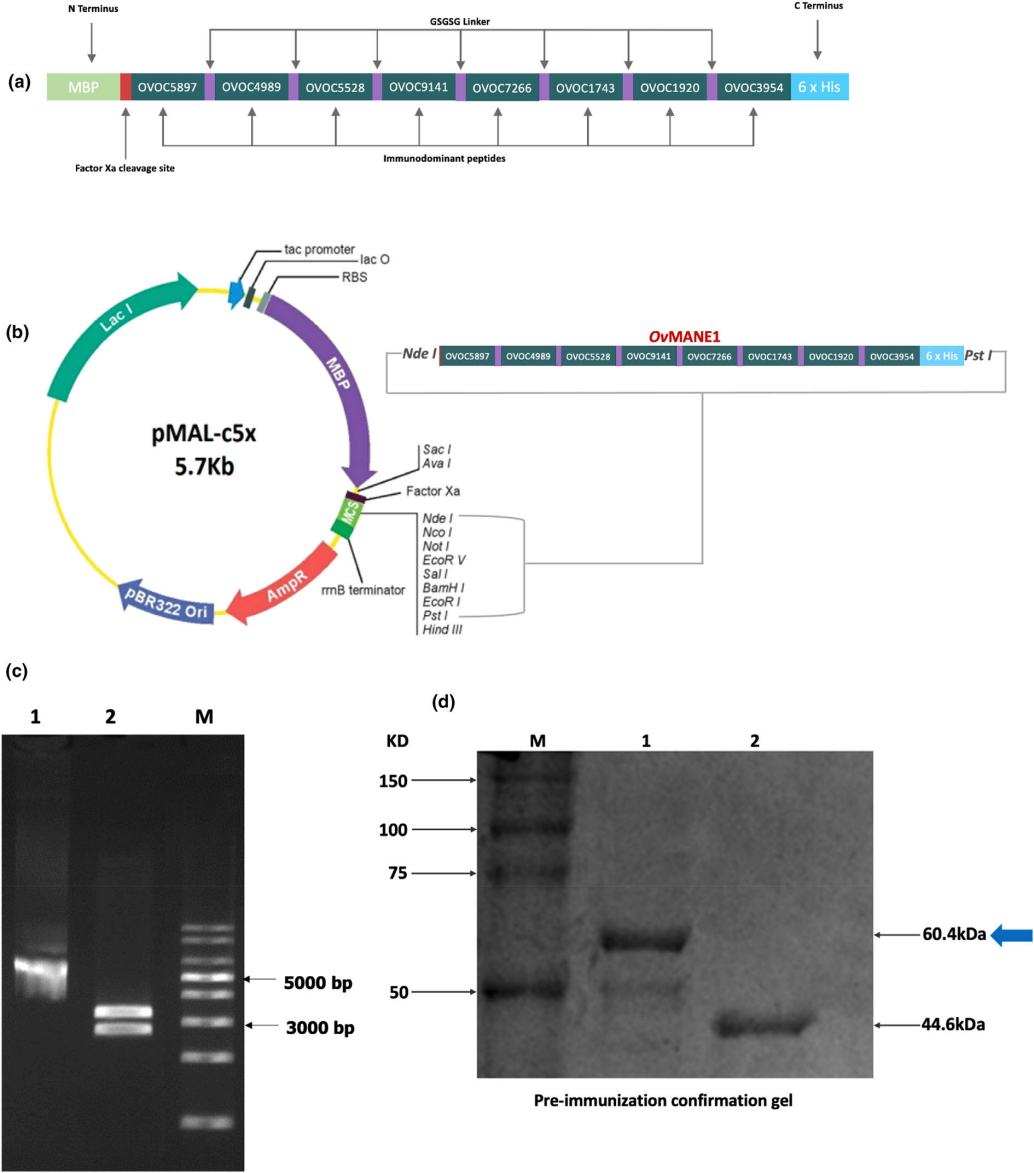
Schematic representation, expression, purification, and detection of *Ov*MANE1. **(a)** Representation of *Ov*MANE1 (560 amino acid residues) consisting of eight immunodominant peptides (coloured navy green) fused with GSGSG linkers (purple stripes) to form the *Ov*MANE1 domain. This domain is linked to an N‐terminal mannose‐binding protein (MBP) (coloured light green) and to a C‐terminal 6x histidine tag (coloured light blue).^20^ **(b)** Expression plasmid map indicating *Ov*MANE1 gene inserted in frame with the MBP‐tag at the N terminus between NdeI and Pst restriction sites. **(c)** Digestion gel of the recombinant pMAL‐c5x plasmid coding for *Ov*MANE1. Expected fragment sizes include for lane 1 = *Ov*MANE1 single digest with MluI – 6096 bp and for lane 2 = *Ov*MANE1 double digest with MluI and HindIII – 3348 bp and 2748 bp. **(d)** SDS‐PAGE confirmation of the integrity of purified *Ov*MANE1 antigen prior to mice immunisation. Lane 1 = *Ov*MANE1 with band at approximately 60.4 kDa, and Lane 2 = MBP band at approximately 44.6 kDa. M: ladder (molecular weights). The blue arrow indicates the positions of *Ov*MANE1.

Previously, we utilised immunoinformatics to design a multiple epitope vaccine candidate, *Ov*‐MANE1 (Figure 1a), which incorporates relevant B‐ and T‐cell epitopes derived from eight immunogenic peptides that were previously evaluated in preclinical studies.^20^ In the process of cloning the chimeric antigen into a pMAL‐c5X vector, an MBP tag was incorporated at the N terminus of *Ov*MANE1 antigen. Addition of the MBP tag enhances protein solubility and stability, improving the expression and purification of resulting fusion antigens, thus facilitating large‐scale pharmaceutical production.^21^ Humoral immune responses to MBP when linked to OvMANE1 chimeric antigen were quite low in previous analyses and could not discriminate between serum samples from onchocerciasis infected and uninfected individuals. This suggests that the immune responses detected to the constituents of recombinant *Ov*MANE1_MBP chimeric antigen were not contributed by the MBP tag.^20^ Overall, *Ov*MANE1 demonstrated remarkable antigenicity, supported by WormBase gene expression data, which confirmed that all peptides incorporated within its full *Ov*‐MANE1 domain are expressed across every stage of the *O. volvulus* parasite.^20^ This underscores its significant potential as both a prophylactic and therapeutic vaccine candidate, offering comprehensive protective coverage throughout the various stages of the *O. volvulus* life cycle.^20^ Consequently, this study aimed at assessing the safety and immune response profile of *Ov*MANE1 + Freund's adjuvant in BALB/c mice model for further characterisation of the antigen as a potential vaccine candidate against onchocerciasis. Freund's adjuvant was chosen for two reasons—affordability and its ability to induce Th1‐biased cellular immunity. The findings demonstrated pronounced production of relevant IgG antibodies and cellular components, including lymphocytes. This strongly suggests that the antigen has the capacity to elicit mixed Th1/Th2 responses and antibody‐dependent cellular cytotoxicity (ADCC) targeting *O. volvulus* L3 and/or other larval stages of the parasite.

## Results

### Expression, purification and confirmation of *Ov*MANE1 chimeric antigen

*Ov*MANE1 chimeric antigen previously designed and reported by us as a promising biomarker for developing a diagnostic test for human onchocerciasis^20^ is made up of eight *O. volvulus* immunodominant peptides derived from the following antigens: OVOC5897, OVOC4989, OVOC5528, OVOC9141, OVOC7266, OVOC1743, OVOC1920 and OVOC3954. The rationally screened immunodominant peptides were fused using flexible GSGSG linkers to enhance the accessibility of the epitopes to antibodies and improve protein folding (Figure 1a). The chimeric antigen was cloned into the pMAL‐c5X vector (Figure 1b), and then successfully expressed and purified as previously reported in our previous study.^20^ WormBase gene expression data indicated that all eight peptides making up the *Ov*MANE1 domain were expressed in all the parasite stages of *O. volvulus*. The addition of an MBP tag to *Ov*MANE1 upon expression in a pMAL‐c5X vector serves to enhance *Ov*MANE1's solubility, stability and large‐scale pharmaceutical production.^21^ Figure 1c shows the digestion gel of the recombinant *Ov*MANE1 pMAL‐c5x plasmid showing the expected fragment sizes resulting from double (3348 and 2748 bp) and single (6096 bp) digestions. Before mice immunisation, the purified antigen was assessed on a 12% polyacrylamide gel to verify its integrity (Figure 1d). As expected, a distinct *Ov*MANE1 band was identified at approximately 60.4 kDa, confirming its integrity for use in downstream mice immunisation experiments.

### Qualitative safety assessment of adjuvanted‐ *Ov*MANE1 antigen

Assessment of adverse effects following immunisation of mice with the adjuvanted‐*Ov*MANE1 antigen primarily involved qualitative monitoring of physical signs of toxicity and behavioural changes after immunisation. Aside from mild erythema (redness of the skin), no other injection site reactions, such as swelling or skin rashes, were noteworthy in any of the groups. Behavioural patterns were also monitored post‐immunisation, with no events such as unusual lethargy or hyperactivity detected. Additionally, eating and drinking behaviours, including appetite and hydration levels, were normal across all groups, and no abnormal social interactions, such as aggression or withdrawal patterns, were observed. These results suggest that *Ov*MANE1 formulated with Freund's adjuvant might be well‐tolerated in BALB/c mice.

### ELISA analysis demonstrates marked IgG antibody responses to adjuvanted‐ *Ov*MANE1

**Figure 2:**
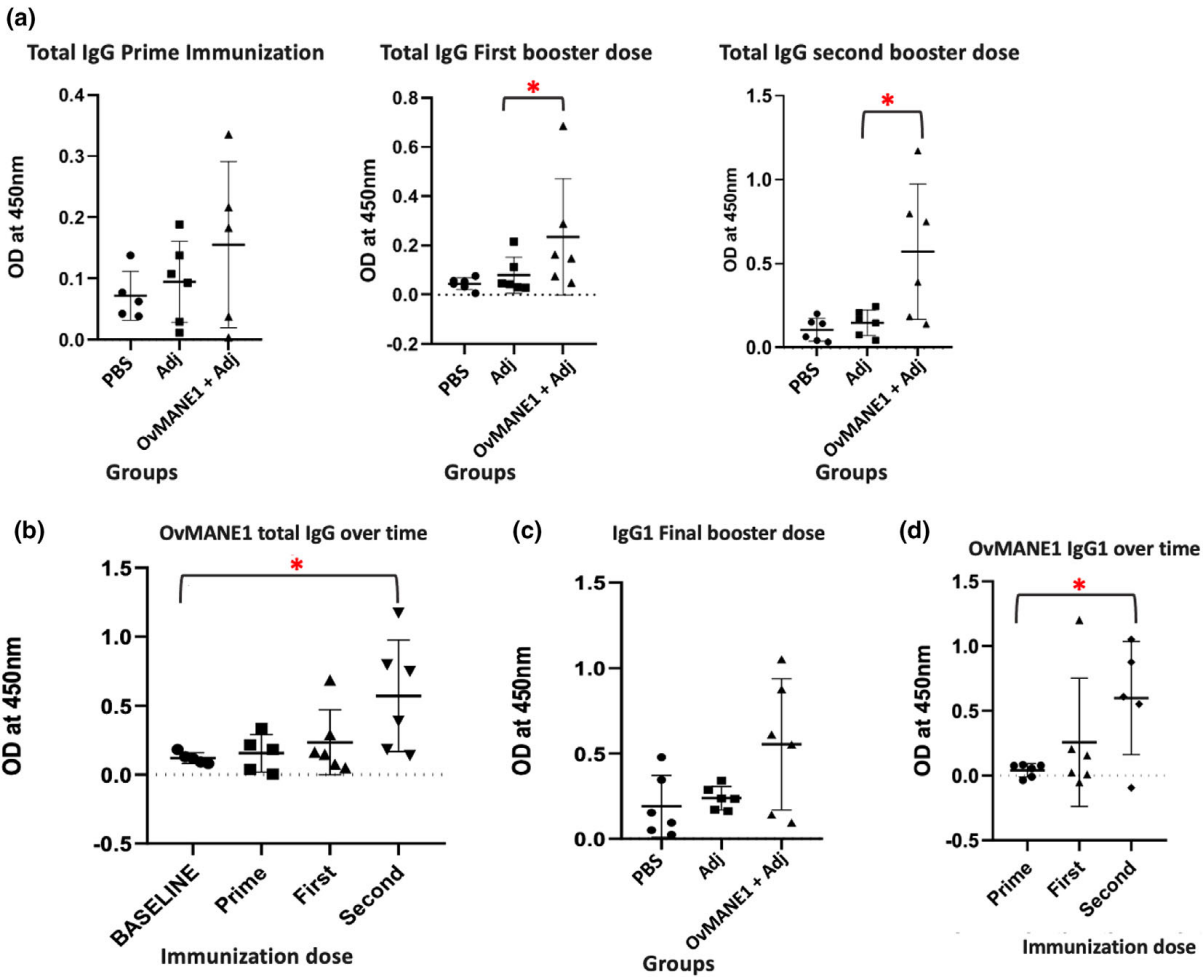
IgG antibody responses to OvMANE1 + Freund's adjuvant relative to control groups and across time. **(a)** Analysis between treatment groups indicating higher absorbance (OD 450 nm) of total IgG in the *Ov*MANE1 + Adjuvant group at all points of immunisation, compared to the controls (PBS and adjuvant‐only groups). **(b)** Analysis within the adjuvanted‐*Ov*MANE1 group showing a significant (*P* = 0.0086) increase in total IgG response over the study period with successive immunisation doses from baseline (Day 0), prime immunisation (Day 11), first boost (Day 25) and second boost (Day 39). **(c)** While IgG1 absorbance were higher in the test group than the controls, the difference was not significant after the final booster dose (Day 39). However, **(d)** analysis within the treatment group indicates a significant difference (*P* = 0.0465) in IgG1 absorbance (OD 450 nm) between the prime and second booster doses. Adj, adjuvant; First, first booster injection; PBS, phosphate buffer saline; Prime, prime immunisation dose; Second, second booster dose. *P‐values <0.05. Experiments were done once.

Measuring total IgG absorbance (OD 450 nm) reflects how effectively the humoral immune system is stimulated by antigens in this context. Previous studies have shown that IgG1 sub‐type specifically plays a crucial role in protecting against parasitic infections.^9^, ^22^ Accordingly, an indirect ELISA was performed to assess total IgG and IgG1 responses to *Ov*MANE1, using sera from immunised mice across the three treatment groups. The findings indicated a significant rise in antibody absorbance (OD 450 nm) on Day 25 following the first boost (day 14; *P* = 0.0260) and on Day 39 after the final boost (Day 28; *P* = 0.026) in the adjuvanted‐*Ov*MANE1 group compared to the adjuvant control group (Figure 2a). Additionally, within the group of mice receiving *Ov*MANE1 formulated with Freund's adjuvant, total IgG absorbance steadily increased over time (Days 0 to 39) with successive immunisation doses, demonstrating a significant (*P* = 0.0086) difference in antibody absorbance between the baseline and post‐final booster dose (Day 39) (Figure 2b). For IgG1, while the adjuvanted‐*Ov*MANE1 group had higher absorbance (OD 450 nm) post‐final booster dose compared to both the adjuvant and PBS control arms, the difference was not significant (*P* = 0.0561) (Figure 2c). Interestingly, a detailed analysis of the *Ov*MANE1 formulated with Freund's adjuvant group revealed a statistically significant increase (*P* = 0.0465) in IgG1 responses over time, demonstrating a consistent upward trend across successive immunisations, from the initial prime dose to the final booster dose (Figure 2d). These findings show clear development of relevant IgG antibodies, suggesting the capacity of the antigen to elicit antibody‐dependent cellular cytotoxicity (ADCC) targeting *O. volvulus* L3 and/or other larval stages of the parasite, a key protective mechanism against onchocerciasis.

### Leukocyte responses indicate that adjuvanted‐ *Ov*MANE1 functions as a potent immunogen

**Figure 3:**
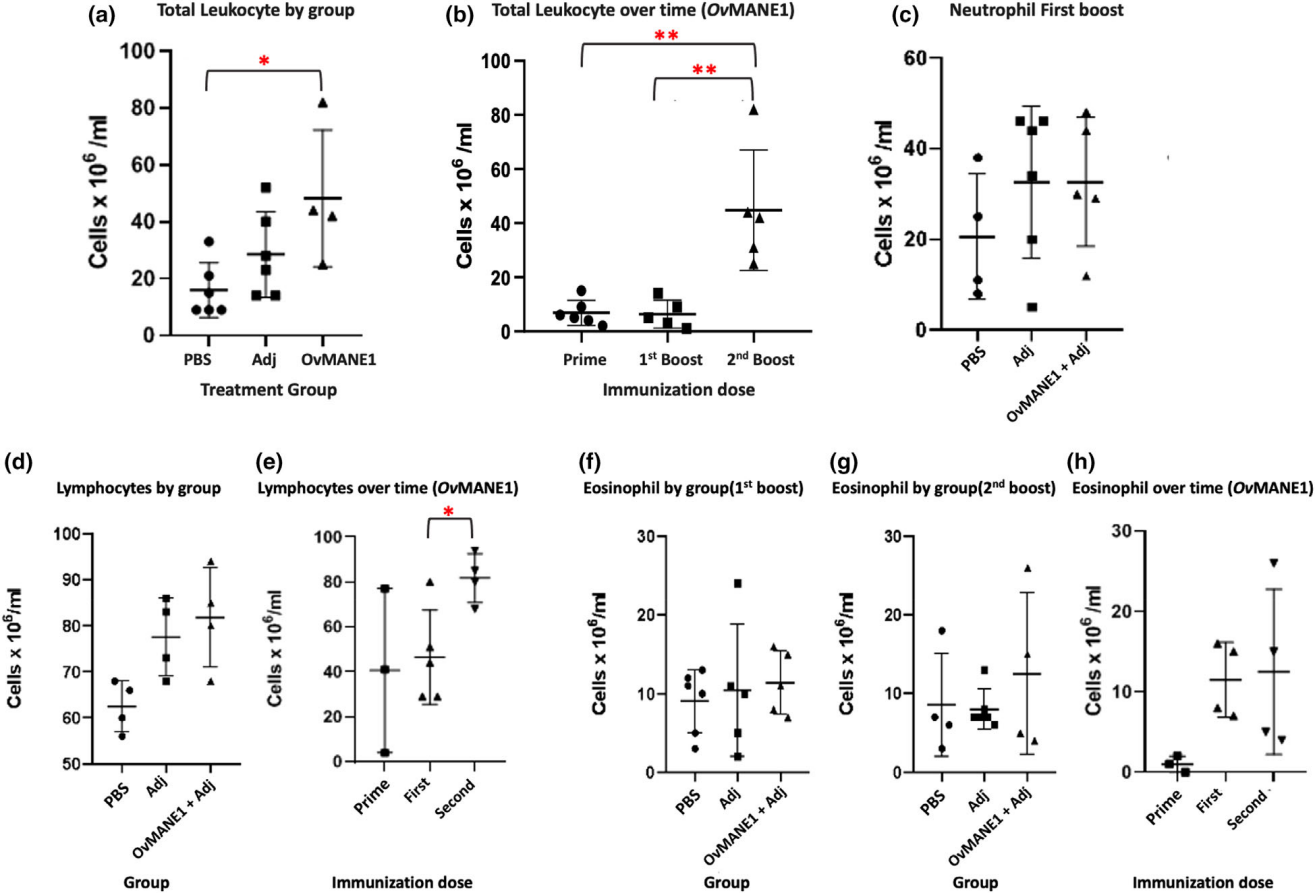
White blood cell responses to adjuvanted‐*Ov*MANE1. **(a)** Total leukocyte responses between treatment groups post‐final booster immunisation dose. A significant increase (*P* = 0.0190) was observed in the adjuvanted‐*Ov*MANE1 group compared to the PBS control group, after the second boost (Day 39). **(b)** Total leukocyte responses within the *Ov*MANE1 + adjuvant group across immunisation time points. A general significant increase in total leukocyte levels was observed in the *Ov*MANE1 group, between the prime (Day 0) and post‐second boost (Day 39, *P* = 0.0043) and between the post‐first booster dose (Day 25) and post‐ second booster dose (Day 39, *P* = 0.0079). **(c)** Neutrophil levels post‐first booster immunisation timepoint for the different treatment groups. The neutrophil levels were higher in the adjuvanted‐*Ov*MANE1 group compared to the PBS control, although not significant. **(d)** Lymphocyte levels post‐final booster immunisation across the various treatment groups. **(e)** Lymphocyte responses within the group of mice immunised with the *Ov*MANE1 formulated with Freund's adjuvant across immunisation time points. A significant increase was observed between the post‐first booster dose and post‐final boost (*P* = 0.0397). Eosinophil responses across the treatment groups **(f)** post‐first booster immunisation dose and **(g)** post‐final immunisation. In both cases, the adjuvanted‐*Ov*MANE1 group exhibited higher eosinophil counts compared to the controls. **(h)** Distribution trend in eosinophil count for the *Ov*MANE1 + adjuvant group across immunisation timepoints with progressive increase from prime dose on day 0 to post‐final immunisation dose on day 39. Adj, adjuvant; First, first booster injection; PBS, phosphate buffer saline; Prime, prime immunisation dose; Second, second booster dose. *P‐values <0.05, **P‐values <0.009. Experiments were done once.

Leukocyte (white blood cells) counts serve as a standard marker for evaluating the cellular immune potential of antigens or adjuvant formulations in experimental studies.^23^ Therefore, to evaluate the cellular responses to *Ov*MANE1 + Freund's adjuvant at different immunisation time points, the total and differential white blood cells count was performed by Giemsa stain microscopy. Overall, a statistically significant rise in total leukocyte count was recorded following the second booster dose administered on Day 28 (*P* = 0.0190) in the adjuvanted‐*Ov*MANE1 group when compared to the PBS control group (Figure 3a), but not significant (*P* = 0.0582) when compared to the adjuvant control. Moreover, in the group of mice immunised with *Ov*MANE1 formulated with Freund's adjuvant, total white blood cell count showed a progressive increase over the study period (Days 0 to 39) with each successive immunisation dose, reflecting significant differences in leukocyte count between the post‐prime dose (Day 0) and post‐final booster dose (Day 39) (*P* = 0.0043); as well as between the post‐first booster dose (Day 25) and post‐final dose (Day 39) (*P* = 0.0079), as illustrated in Figure 3b.

To further determine the specific cellular responses to the adjuvanted‐*Ov*MANE1, differential leukocyte levels, with a focus on neutrophils, lymphocytes and eosinophils, were evaluated among the different treatment groups at different immunisation time points. The result showed that the *Ov*MANE1 + Adjuvant group had more neutrophils (Figure 3c) and lymphocytes (Figure 3d) induced than the PBS and adjuvant control groups post‐final (Day 39) immunisation, although the differences between the test and control groups were not significant. However, the data indicated a significant increase (*P* = 0.0397) in lymphocyte counts within the adjuvanted‐*Ov*MANE1 treatment arm over time, between the post‐first booster dose (Day 25) and post‐final booster dose (Day 39) as shown in Figure 3e.

Furthermore, eosinophil responses were evaluated across the different treatment groups and across successive immunisations over time. The results indicate that the adjuvanted‐*Ov*MANE1 group exhibited higher eosinophil counts following both the first (Day 25) (Figure 3f) and second (Day 39) (Figure 3g) booster doses compared to the control groups, although the differences were not statistically significant in either case. Likewise, for the mice immunised with *Ov*MANE1 formulated with Fruend's adjuvant, sharp and mild elevations in eosinophil levels were noted between the post‐prime (Day 11) and post‐first (Day 25) booster dosing periods, as well as between the post‐first (Day 25) and post‐second (Day 39) booster dosing intervals, respectively, over the study timeline. However, these increases were not significant (Figure 3h). Nonetheless, these findings collectively demonstrate that adjuvanted‐*Ov*MANE1 elicits a strong and sustained leukocyte‐mediated immune response, underscoring its potential as a promising immunogen.

## Discussion

River blindness continues to pose a major challenge in developing countries such as Cameroon, where community‐directed treatment with ivermectin, the main control tool against the infection, faces notable limitations such as ivermectin resistance and the lack of macrofilaricidal drugs.^8^, ^24^ As a result, there is an urgent need for alternative intervention strategies, including prophylactic vaccines to combat the disease, supporting the WHO's 2030 global onchocerciasis elimination agenda.^25^ Our research, thus, aimed to evaluate the capability of a novel multi‐epitope chimeric antigen, *Ov*MANE1, to safely elicit suitable humoral and cellular immune responses following immunisation of BALB/c mice at three different time points of two‐week intervals. The assessment of adjuvanted‐*Ov*MANE1 immunisation in mice revealed no significant physical signs of toxicity nor behavioural abnormalities, suggesting its promising safety profile and suitability for clinical development. On the other hand, analysis of the humoral responses demonstrated a significant increase in total IgG and IgG1 antibody absorbance (OD 450 nm) in the adjuvanted‐*Ov*MANE1‐immunised group compared to controls (Figure 2). Similarly, whole and differential leukocyte analyses reveal that *Ov*MANE1 + adjuvant induced a strong and sustained cellular immune response in mice, underscoring its potential as an effective immunogen (Figure 3).

*Ov*MANE1 recombinant chimeric antigen was successfully designed and produced from synthesis of the conceptual gene construct through expression to purification, as previously described in our previous study.^20^ Similar approaches using multi‐epitope chimeric antigens have demonstrated strong immunogenic potential in other studies, such as in phase I clinical trials targeting *Plasmodium falciparum* antigens,^26^, ^27^ thus recommending the promising profiles of such multi‐peptide vaccine candidates. The design of *Ov*MANE1 incorporated an MBP motif at its N terminus to improve the antigen's solubility and stability, with the goal of simplifying and enabling effective large‐scale pharmaceutical manufacturing^21^ while facilitating the targeted delivery of the *Ov*MANE1 segment to antigen‐presenting cells (APCs), thereby driving strong Th1 responses and cytotoxic T‐cell activation.^28^, ^29^ Adjuvanted‐*Ov*MANE1 demonstrated marked antigenicity in enzyme‐linked immunosorbent assays. A key strength of the antigen relies on the fact that it consists of eight immunodominant epitopes expressed across all the life cycle stages of the *O. volvulus* parasite,^20^ emphasising its potential as both a prophylactic and therapeutic vaccine against river blindness.

Indeed, mice immunisation with *Ov*MANE formulated with Freund's adjuvant was well‐tolerated, as we observed no physical signs of toxicity and behavioural changes including lethargy, hyperactivity and abnormal social interactions. Nonetheless, the mild erythema observed at mice injection sites across all treatment groups is consistent with immune activation responses typically associated with tissue injury because of injection site skin damage, as well as antigen‐adjuvant combinations, as reported by Kool *et al*.^30^ However, the stable appetite and hydration levels witnessed across all mice groups throughout the study duration further strengthen the case for *Ov*MANE1 as a safe antigen for clinical development.

*Ov*MANE1 also induced potent antibody responses, as evidenced by a significant rise in total IgG antibody absorbance (OD 450 nm) following the first (*P* = 0.0260) and second (*P* = 0.0260) booster doses (Figure 2a and b), compared to adjuvant control. Similarly, the gradual increase in antibody absorbance over time, equally, underpins the effectiveness of Freund's adjuvanted‐*Ov*MANE1 in stimulating sustained humoral immune activation. These findings align with previous research illustrating that multi‐epitope antigens can elicit strong and durable humoral immunity in murine models.^13^, ^27^ The significance of total IgG and IgG1 in protecting against onchocerciasis has been highlighted^11^ and supports the relevance of the elevated IgG and IgG1 antibody responses to *Ov*MANE1 detected herein. Therefore, the significant and steady increase in total IgG (*P* = 0.0086) and IgG1 (*P* = 0.0465) absorbance observed between the baseline and final booster immunisations further supports the capacity of the adjuvanted‐*Ov*MANE1 to prime and enhance humoral immunity, making it a promising candidate for onchocerciasis vaccine development. These results correspond with numerous studies highlighting the cytophilic effect of IgG antibodies against *O. volvulus* parasites in both murine and non‐human primate immunisation challenge experiments.^9^, ^11^

Our findings also indicate that *Ov*MANE1 + adjuvant effectively induces cellular immune responses, as the total leukocyte counts following booster immunisations significantly increased (*P* = 0.0190) than the control (Figure 3a). This observation corresponds with prior research demonstrating similar patterns of leukocyte proliferation in response to protective filarial antigens.^31^, ^32^ The progressive rise in total white blood cell counts throughout the immunisation schedule, with significant differences between first‐boost and post‐final booster (*P* = 0.0043) (Figure 3b), suggests successful immune priming and memory response induction and so aligns with similar findings from other recombinant helminth vaccines.^33^ Although neutrophil and lymphocyte proliferation in mice immunised with adjuvanted‐*Ov*MANE1 was not significantly elevated compared to the controls, notable increases were observed relative to the PBS control (Figure 3c and d). Moreover, lymphocyte responses within the *Ov*MANE1 + adjuvant group demonstrated significant temporal elevations (*P* = 0.0397) (Figure 3e), and eosinophil counts exhibited marked increases following booster doses (Figure 3f–h). However, the absence of statistically significant differences in eosinophil levels compared to the control groups warrants further investigation. These trends are consistent with observations from promising vaccine candidates for related filarial parasites,^32^, ^33^ where early cellular responses preceded stronger adaptive immunity. The sustained leukocyte‐mediated immune activation elicited within the adjuvanted‐*Ov*MANE1‐immunised mice group might present *Ov*MANE1 as a potential vaccine candidate against human onchocerciasis, though additional research on long‐term immunity and protective efficacy is required to confirm its full prospects.

**Figure 4:**
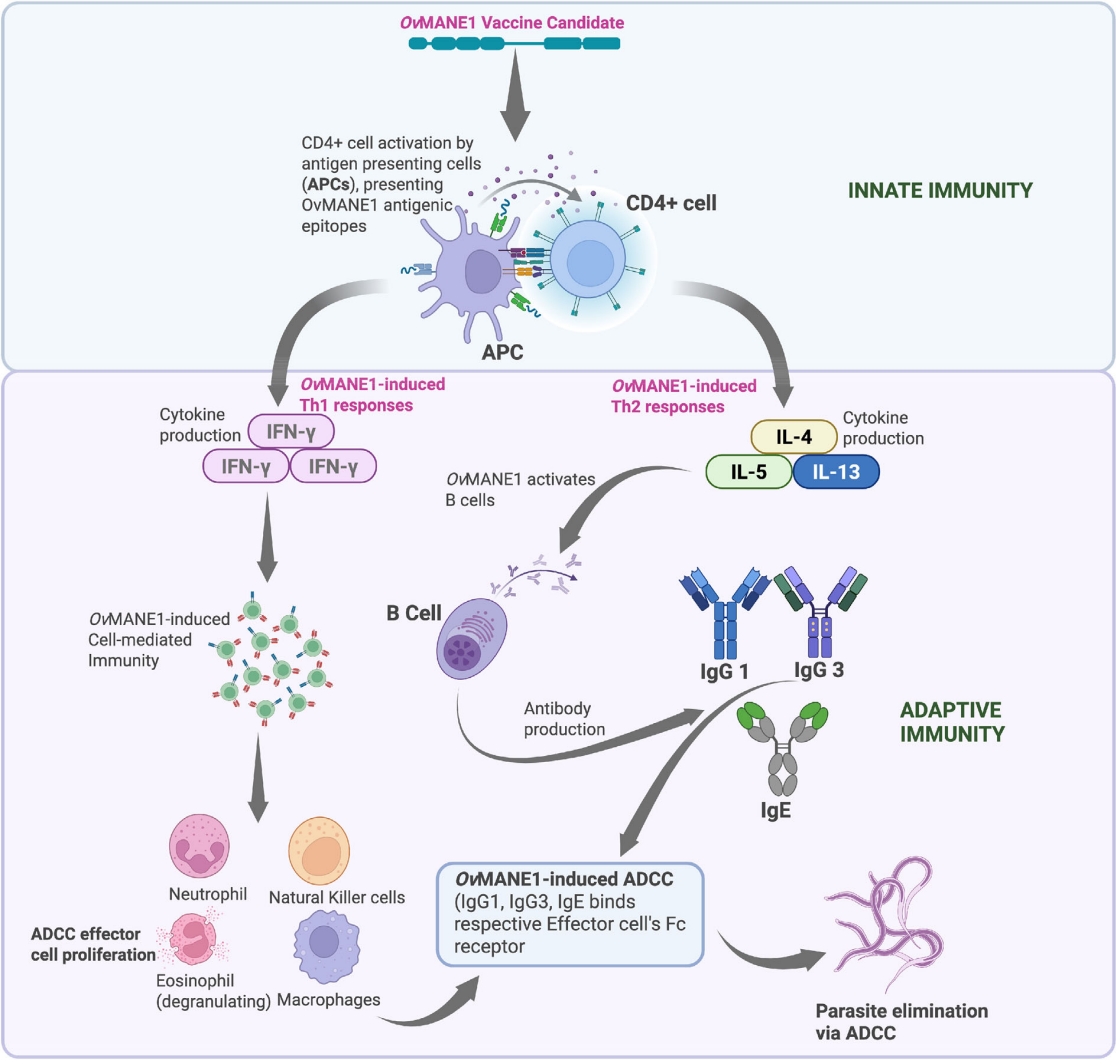
Ideal antigen‐induced protective immune mechanisms against onchocerciasis. The upper section highlights the activation of antigen‐presenting cells (APCs), which process and present antigenic epitopes to CD4+ T cells (lymphocyte), inducing distinct Th1 and Th2 responses. Antigen‐induced Th1 responses would potentially stimulate cytokine production, particularly interferon‐gamma (IFN‐γ), which supports cell‐mediated immunity and activates effector cells (neutrophils and eosinophils with marked proliferation observed herein), perhaps contributing to antibody‐dependent cellular cytotoxicity (ADCC) through expression of their respective families of Fc receptors for antibody binding. In contrast, potential antigen‐induced Th2 responses would drive the production of interleukins such as IL‐4, IL‐5, and IL‐13, promoting humoral immunity and B‐cell activation. Through adaptive immunity, B cells then produce immunoglobulins (total IgG and IgG1 with marked increases in absorbance (OD 450 nm) as observed in this study) in response to Th2 cytokine stimulation. These antibodies might bind the effector cells via Fc receptors to complete the ADCC pathway and enhance parasite elimination.

Certain mechanisms underlying acquired protective immunity against *O. volvulus* infection in humans (Figure 4) have been examined, and so revealed that protection is linked to the ability to elicit mixed Th1/Th2 responses targeting *O. volvulus* L3 and/or other larval stages of the parasite.^34^, ^35^ Furthermore, effective anti‐helminth immunity through antibody‐dependent cell‐mediated cytotoxicity (ADCC) against L3 is facilitated by cytophilic IgG antibodies including IgG1 and IgG3; as well as cytokine release.^10^, ^34^ In this research, we recorded marked yet consistent increases in total IgG absorbance (OD 450 nm) and significant IgG1 absorbances (*P* = 0.0465) (Figure 2), along with proliferation of overall cellular responses, including a significant rise in lymphocytes over time and a notable rise in neutrophil and eosinophil counts when comparing the adjuvanted‐*Ov*MANE1‐vaccinated mice group to the PBS control (Figure 3).

Consequently, this evident formation of IgG antibodies and cellular elements could mean that *Ov*MANE1 induces the necessary components needed to elicit antibody‐dependent cellular cytotoxicity (ADCC) (Figure 4). However, functional ADCC assays are needed to confirm this. Indeed, earlier studies suggest that neutrophils and eosinophils can facilitate ADCC through Fc receptor expression, thereby amplifying their cytotoxic efficacy,^36^, ^37^ while also accentuating their involvement in ADCC. Additionally, the significant proliferation of T lymphocytes (Figure 3e) contributes to anti‐helminth immunity through cytokine production (particularly Th2 responses including IL‐4, IL‐5 and IL‐13) which supports eosinophil function and antibody production. Increased total IgG1 absorbance (OD 450 nm) is indicative of a Th2‐skewed response and associated cytokines like IL‐4 and IL‐5, aligning with findings by Du *et al*.,^38^ while lymphocyte proliferation upon immunisation suggests overall immune activation, indicative of proliferation of Th1 cells which serve as key drivers of cellular immunity and are associated with cytokines like IFN‐γ.^39^

Despite these encouraging data, our study is not without limitations. For instance, the research was restricted to murine models, which might not fully represent the intricate immune responses seen in humans. The other main limitation is the fact that we only conducted this study once in mice, and the experiments need to be repeated to confirm the results obtained. However, this is the first time the *Ov*MANE1 antigen is tested in mice, and so this study focussed more on setting up a pipeline for testing the antigen in rodents using local equipment in a third‐world laboratory. Follow‐up studies are underway using the optimised version of this protocol and will be repeated in different mice experiments to confirm the *Ov*MANE1‐induced responses. We also recognise Freund's adjuvant's unsuitability for human vaccines because of toxicity and regulatory restrictions. However, its use was to establish proof‐of‐concept immunogenicity and was motivated by affordability and its well‐documented ability to elicit strong Th1 biased responses in preclinical models. It provided a baseline comparator for immune profiling. Follow‐up studies screening clinically acceptable adjuvants, including Alum and CpG‐based formulations, should be conducted to validate whether similar immune responses can be achieved safely.

Furthermore, a key methodological limitation arose from the reliance on light microscopy to evaluate cellular immunity to the antigen. This approach introduced some degree of subjectivity in visual interpretation, which could have resulted in confirmation bias. Additionally, differential white blood cell counts were performed by manually counting at least 100 leukocytes per slide. Given the relatively low frequency of eosinophils in peripheral blood, this counting density may provide limited sensitivity for detecting subtle eosinophil changes. This could be a reason for the negative eosinophil results observed. However, flow cytometry, antigen recall assays, T‐cell phenotyping and a functional immune assay could be used in subsequent studies to help strengthen the analysis. The method was also unable to distinguish between CD4+ and CD8+ cells. Moreover, consistent blood sampling of mice using standard retro‐orbital bleeding protocol proved challenging. Consequently, concerns about potential harm to the animals and limitations associated with capillary tube blood clotting rendered it impractical to collect samples from some mice at the predefined time points. This limitation resulted in missing data points for antibody and cellular responses for some mice across treatment groups. Another limitation of this study is the relatively short antibody follow‐up. IgG responses were monitored only until Day 39 post‐immunisation. This follow‐up period may be insufficient to assess the long‐term durability of vaccine‐induced humoral immunity, particularly in the context of infection with onchocerciasis which is a chronic parasitic condition. We would, therefore, interpret the short‐term serologic increases cautiously and recommend further follow‐up studies to measure antibody persistence at 6 and 12 months. Additionally, in prior analyses, the humoral immune responses directed against MBP linked to *Ov*MANE1 were minimal and failed to differentiate serum samples from onchocerciasis infected vs. uninfected individuals.^20^ These findings suggest that the observed immune recognition of the recombinant *Ov*MANE1_MBP antigen components was unlikely attributable to the MBP fusion tag, but largely to the *Ov*MANE1 component. Nevertheless, future research employing advanced techniques (e.g. blood sampling and flow cytometry) are planned to achieve a more precise and detailed characterisation of immune responses to *Ov*MANE1 alone, without the MBP tag. Furthermore, less toxic adjuvants such as Alum are recommended for use in future animal challenge studies to mitigate the likelihood of toxicity accompanying the use of Freund's adjuvant.

In summary, our study provides strong evidence underpinning the potential of the novel multi‐epitope chimeric antigen, *Ov*MANE1, as a promising vaccine candidate against onchocerciasis. Immunisation of BALB/c mice elicited significant increases in both humoral and cellular immune responses, including evident increases in total IgG and IgG1 antibody absorbance (OD 450 nm), as well as notable leukocyte proliferation, particularly involving lymphocytes, neutrophils and eosinophils. These findings suggest that *Ov*MANE1 could effectively protect against *O. volvulus*, hence contributing to the World Health Organization's 2030 goal of onchocerciasis elimination. Future investigations could include detailed studies in other small animal species^40^ and non‐human primates to assess the safety, immunogenicity, and efficacy of *Ov*MANE1 in much more detail and context. Additionally, data from animal challenge experiments with *O. volvulus* or sister species such as *O. ochengi* parasites are needed to ascertain the protective immune mechanisms of *Ov*MANE1. Understanding the durability of the induced immunity and elucidating the mechanisms driving the immune response to this antigen will be crucial for its clinical development and use.

## Methods

### Ethical consideration for animal studies

Ethical clearance for the use of mice models was obtained from the University of Buea Institutional Animal Care and Use Committee (IACUC) with reference number (UB‐IACUC No. 02/2021). The validated ethical consideration protocols were adhered to throughout the study to ensure the humane treatment of the animals.

### Animal model and grouping

This study involved BALB/c mice purchased from the Jackson Laboratory (Bar Harbor Maine, USA), aged 6–8 weeks, weighing approximately 16–27 g (Table 1). Mice were housed in micro‐isolator boxes in standard laboratory conditions (temperature: 23 ± 2°C and 12‐h light/dark cycle) and provided with *ad libitum* access to food and water. The mice were randomly assigned to three treatment groups: (1) control group receiving phosphate‐buffered saline (PBS), (2) adjuvant control group receiving Adjuvant + PBS, and (3) test group receiving *Ov*MANE1 antigen + Adjuvant + PBS (Tables 2 and 3). Each group consisted of 6 mice. All protocols involving mice were approved by the Institutional Animal Care and Use Committee of the University of Buea.

**Table 1:** Mice body weight and grouping

Sex	Cage no.	ID	Groups	Body weight (g)
M	1	0	PBS	22
M	1	1	Adjuvant	16.3
M	1	2	OV‐MANE	20
M	1	3	PBS	22
M	1	4	Adjuvant	16.3
M	1	5	PBS	26.1
M	2	2	OV‐MANE	24.3
M	2	0	PBS	21.4
M	2	1	Adjuvant	22.3
M	2	3	OV‐MANE	22.2
F	2	4	PBS	21.9
F	2	5	PBS	22.2
F	3	0	Adjuvant	20.9
F	3	1	Adjuvant	21.8
F	3	2	Adjuvant	22
F	3	3	OV‐MANE	25.5
F	3	4	OV‐MANE	21.8
F	3	5	OV‐MANE	18.9

PBS, phosphate buffered saline.

**Table 2:** Mice immunisation treatment groups

Group no.	Group name	Treatment
1	Control group	Phosphate buffered saline (PBS)
2	Control group	PBS + Adjuvant
3	Test group	PBS + Adjuvant + *Ov*MANE1

**Table 3:** Distribution of volume of immunisation doses per mice group

Mice group number	Treatment arms	*Ov*MANE1	Adjuvant	PBS	Total volume
1	PBS control	—	—	150 μL	150 μL
2	PBS + Adjuvant control	—	1 mg (75 μL)	75 μL	150 μL
3	PBS + Adjuvant + *Ov*MANE1	25 μg (11.1 μL) stock: 2.25 mg mL	1 mg (75 μL)	63.9 μL	150 μL

PBS, phosphate buffered saline.

### Collection of mice baseline data

The individual weights of the mice were measured using an electronic weighing scale and mice were tagged by serial numbering from one to 18. Blood samples were collected through retro‐orbital bleeding following standard protocol^41^ into 0.5‐mL EDTA‐coated microcentrifuge tubes for preliminary whole leucocyte and differential cell count. Blood samples were aliquoted into dry 0.5‐mL Eppendorf tubes, centrifuged at 13 250 *g* for 15 min, and serum collected into sterile 0.5‐mL Eppendorf tubes and stored at −20°C for downstream serological analysis.

### Mice immunisation

**Figure 5:**
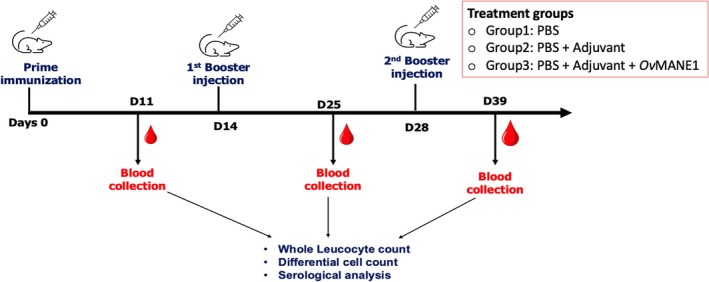
Mice immunisation and sample collection plan. BALB/c mice were subcutaneously immunised thrice at 2‐week intervals. Whole blood was collected by retro‐orbital bleeding at various time points for whole leucocyte counts, differential cell counts and serological analysis. D11‐39 represents Days 11 to 39. PBS: vehicle negative control group. Adjuvants were Freund's complete adjuvant for prime dose and Freund's incomplete adjuvant for booster doses.

The total antigen and vehicle volumes for immunisation were prepared per group based on the description on Table 3. The solutions were prepared for three injection doses; priming, first boost and second booster injections following standard protocol.^41^ Freund's complete adjuvant (CFA) was used for the priming, while Freund's incomplete adjuvant (IFA) was used for the two booster injection doses with antigen and vehicle volumes as described in Table 3. To prepare the adjuvanted antigen solution, CFA or IFA adjuvants were added to the *Ov*MANE1 antigen solution (cloned, expressed and purified as previously reported by us^20^) and the mixture vortexed. After an eight‐day mice acclimatisation period, a 25 gauge syringe was used to deliver a 150 μL injection containing respective treatment doses to each mouse. Injections were administered subcutaneously at the nape. The initial prime dose was administered on day 0, followed by two booster doses on days 14 and 28 (Figure 5). One hundred microlitres of blood was collected by retro‐orbital bleeding using standard protocols^41^ at baseline and on Days 11, 25 and 39 post‐immunisation for immunological assays. Additionally, serum samples were obtained at similar time points for serological analysis (Figure 5). Qualitative assessment of adjuvanted‐*Ov*MANE1's safety was performed post‐immunisation. Mice were observed qualitatively for physical and behavioural signs of toxicity and adverse side effects *ad libitum* over the duration of the study. Behavioural monitoring encompassed observations of activity levels (checking for lethargy or hyperactivity), feeding and drinking patterns, as well as social interactions among the mice including potential aggressive behaviour or withdrawal patterns.

### Whole leukocyte and differential white blood cell count

The whole leukocyte count was performed to provide an overview of the general immune cell response to *Ov*MANE1. Turk's fluid was prepared locally with Gentian violet, acetic acid and distilled water according to the standard preparation protocol^42^ and blood samples previously collected in EDTA‐coated tubes were diluted into Turk's solution in a 1:20 ratio. A drop of the mixture was then loaded on a haemocytometer and mounted under a 40x objective light microscope lens and observed according to standard leukocyte enumeration protocol. Leukocytes were counted and recorded using the formula:

$$\text{Whole leukocytes}\;\mathrm{per}\;\mathrm{mL}=\frac{\text{Cells counted}\;x\;10000}{\text{Number of large squares}}\;\times \;20$$

To understand the specific components of the immune responses, differential white blood cell count was also performed. About 5 μL of mice EDTA‐whole blood samples were used to prepare a thin blood smear on microscopic slides and stained with 10% Giemsa using standard Giemsa staining protocol (S. S. College, Jehanabad's protocol of Enumeration of WBC—Total Leukocyte Count (TLC)). Differential white blood cell count was performed manually by examining slides under a 100× oil immersion light microscope, counting at least 100 white blood cells. Lymphocyte neutrophils, and eosinophil cell types were identified and differentially classified.

### Antibody detection by enzyme‐linked immunosorbent assay

The ELISA protocol used by Arumugam *et al*.^43^ with slight modification was used to detect, in duplicates, the presence of antibodies (IgG and IgG1) induced by *Ov*MANE1 in the serum of immunised mice. Briefly, MaxiSorp™ 96‐well microtiter plates (Nunc, Roskilde, Denmark) were coated with 50 μL perwell of 2 μg mL^−1^ of *Ov*MANE1 in 0.5 m carbonate buffer (pH 9.4) at 4°C overnight. Plates were then washed thrice with 0.1% PBS‐Tween‐20 (PBS‐T) after a 5 min interval and blocked with 200 μL per well of 3% non‐fat milk for 2 h at room temperature. The plates were then washed thrice with PBS‐T, and individual serum samples diluted at 1:27 000 in 1% non‐fat milk and 50 μL serum samples added to corresponding wells. Plates were incubated at room temperature for 2 h and washed thrice with PBS‐T. Goat anti‐human peroxidase conjugated IgG (Merck Millipore, Billeria, MA) was prepared in a 1% non‐fat milk solution at a 1:5000 dilution, and 50 μL was added into each well. Plates were incubated for an hour before being washed thrice again with PBS‐T. Finally, 50 μL of the TMB‐KPL substrate was added to each well and the colour was allowed to develop for 10 min at room temperature. The reaction was stopped by adding 50 μL of 3 m HCl per well. The optical densities were recorded using a microplate reader at 450 nm and antibody absorbance estimated. All washing and antibody dilutions were done in the wash buffer (0.1% PBS‐Tween‐20).

### Statistical analysis

All data were recorded and processed using Microsoft Excel 2013. Statistical analyses were performed with the GraphPad Prism 8.0 software (GraphPad Software, La Jolla, CA, USA). Antibody absorbance (OD 450 nm) was compared using the two‐tailed Mann–Whitney *t*‐test for nonparametric data analysis. ANOVA (Analysis of Variance) was used to identify significant differences between groups, while a *P*‐value < 0.05 was considered the threshold of statistical significance for all parameters, and at 95% confidence interval.

## Author contributions

**Derrick Neba Nebangwa:** Conceptualization; methodology; software; data curation; resources; formal analysis; project administration; validation; visualization; writing – review and editing; writing – original draft; funding acquisition; investigation. **Stephen Mbigha Ghogomu:** Conceptualization; methodology; investigation; supervision; writing – review and editing; project administration. **Mary Teke Efeti:** Methodology; data curation; formal analysis; investigation; visualization; writing – original draft; writing – review and editing. **Fidele Ntie‐Kang:** Software; methodology; supervision; project administration; resources; writing – review and editing. **Sonia M. E. Momnougui:** Methodology; data curation; formal analysis; investigation; visualization; writing – review and editing. **Rene Bilingwe Ayiseh:** Methodology; investigation; formal analysis; supervision; writing – review and editing; visualization; project administration. **Robert Adamu Shey:** Methodology; formal analysis; investigation; writing – review and editing; supervision; resources. **Cabirou Mounchili Shintouo:** Methodology; investigation; formal analysis; writing – review and editing; visualization.

## Conflict of interest

The authors declare no conflict of interest.

## Ethics statement

Use of mice and the experimental procedures performed in this study were reviewed and approved by the IACUC committee of the University of Buea, Cameroon.

## Data Availability

The data that support the findings of this study are available from the corresponding author upon reasonable request.
